# 

**Published:** 1899-07

**Authors:** 


### CONTENTS.

ORIGINAL COMMUNICATIONS—

APPENDICITIS: FROM THE STANDPOINT BF A COUNTRY DOCTOR. By A. C. Davidson, M.D., Sharon, Ga .. 289

HOW CAN THE SPREAD OF SCARLET FEVER AND DIPHTHERIA BE PREVENTED IN PUBLIC SCHOOLS? By Gilman Robinson, M.D., Atlanta, Ga .......... 30[illegible]

THE NECESSITY OF A STATE PEDIATRIC SOCIETY. By Oliver B. Bush, M.D., Colquitt, Ga .......... 30[illegible]

A CASE OF CHRONIC PACHYMENINGITIS FOLLOWING THE COURSE OF THE SUPERIOR LONGITUDINAL SINUS, AND APPARENTLY THE RESULT OF CHRONIC SYPHILITIC RHINITIS. By Eugene R. Corson, M.D., Savannah, Ga .......... 30[illegible]

NOTE ON THE TREATMENT OF MALIGNANT NEOPLASMS BY ELECTRICITY. By J. McF. Gaston, Jr. Atlanta, Ga .......... 31[illegible]

SOCIETY REPORTS—

ATLANTA SOCIETY OF MEDICINE .......... 31[illegible]

EDITORIAL NOTES AND COMMENTS .......... 3[illegible]

NEWS AND NOTES .......... 33

CONTENTS CONTINUED .......... v

